# Corneal nerve fiber morphology following COVID-19 infection in vaccinated and non-vaccinated population

**DOI:** 10.1038/s41598-024-67967-x

**Published:** 2024-07-22

**Authors:** Eszter Szalai, Katalin Nagy, Zsofia Kolkedi, Adrienne Csutak

**Affiliations:** Department of Ophthalmology, University of Pécs Medical School, Rákóczi U. 2, Pécs, 7623 Hungary

**Keywords:** Corneal nerves, COVID-19, Dendritic cell, Neuroprotection, Vaccination, Viral infection, Biomarkers

## Abstract

To examine corneal subbasal nerve changes in patients who received vaccination against SARS-CoV-2 virus and underwent COVID-19 infection compared to infected non-vaccinated patients and healthy controls. Twenty-nine eyes of 29 vaccinated patients (mean age: 36.66 ± 12.25 years) within six months after PCR or Ag test proven COVID-19 infection and twenty-eight eyes of 28 age-matched infected, non-vaccinated patients (mean age: 42.14 ± 14.17 years) were enrolled. Twenty-five age-matched healthy individuals (mean age: 47.52 ± 18.45 years) served as controls. In vivo confocal microscopy (Heidelberg Retina Tomograph II Rostock Cornea Module, Germany) was performed in each group. Corneal subbasal nerve plexus morphology and corneal dendritic cells (DC) were evaluated. Significantly higher corneal nerve fiber density (P < 0.001), nerve branch density (P < 0.001), nerve fiber length (P < 0.001), total branch density (P = 0.007), nerve fiber area (P = 0.001) and fractal dimension (P < 0.001) values were observed in vaccinated patients after COVID-19 infection compared to the non-vaccinated group. Significantly higher DC density was observed in the non-vaccinated group compared to the control group (P = 0.05). There was a statistically significant difference in the size of mature DCs (P < 0.0001) but the size of immature DCs did not differ significantly among the 3 groups (P = 0.132). Our results suggest that SARS-CoV-2 vaccination may have a protective effect against the complications of COVID-19 disease on the corneal subbasal nerve fibers.

## Introduction

COVID-19 has been associated with a range of neurological complications. A large cohort study reported neurological manifestations in 80% of COVID-19 patients including central nervous system (CNS) manifestations (dizziness, headache, impaired consciousness, acute cerebrovascular disease, and epilepsy), peripheral nervous system (PNS) manifestations (anosmia, hypogeusia, visual impairment, and neuralgia), and skeletal muscular damage^1^. These complications are thought to occur due to a combination of direct viral invasion of the nervous system and the immune response to the virus^2^.

In vivo confocal microscopy (IVCM) is a non-invasive imaging technique that allows for high-resolution imaging of the corneal layers and subbasal nerve plexus. It has been increasingly used not only in ophthalmology but also in the study of systemic diseases due to its potential to provide insights into the peripheral nerves and cellular changes associated with various systemic conditions. Corneal cellular and nerve fiber changes after COVID-19 diseases have previously been investigated with IVCM^3–5^. Previous authors reported corneal neuroinflammatory alterations in patients after a wide spectrum of COVID-19 disease severity^6,7^.

Multiple types of vaccines against COVID-19 have been developed and are now being utilized worldwide. Vaccines are primarily based on the SARS-CoV-2 spike protein and have been generated by various technologies including messenger RNA (Pfizer-BioNTech and Moderna)^8,9^, adenoviral vectors (AstraZeneca, Janssen and Sputnik)^10–12^. or inactivated virus (Sinopharm and Sinovac)^13^. It is still unclear whether these vaccines prevent viral spread to the CNS and provide protection against the brain damage induced by the virus infection leading to neuroprotective effects^14^.

The purpose of the present study was to examine corneal subbasal nerve morphology in patients who received vaccination against SARS-CoV-2 virus and underwent COVID-19 infection compared to infected non-vaccinated patients and healthy controls.

## Patients and methods

This study involved 82 participants at the Department of Ophthalmology, University of Pecs. Twenty-nine previously vaccinated patients within 6 months of PCR or Ag test proven COVID-19 infection (VCoV group), 28 non-vaccinated patients after COVID-19 infection (NVCoV group) and 25 age-matched healthy controls were enrolled. Control individuals were chosen among individuals who arrived for regular eye examinations and had no prior record of systemic illnesses such as diabetes mellitus, rheumatic conditions, or connective tissue disorders. Healthy controls were not vaccinated against COVID-19 and none of the control participants ever had COVID-19 infection. Those who had experienced SARS-CoV-2 infection were omitted from the study if they possessed any pre-existing systemic conditions, which encompassed metabolic and cardiovascular ailments. In the three groups, there were no instances of previous or ongoing ocular disorders, utilization of contact lenses, or intraocular surgery.

The study was performed in accordance with the tenets of the Helsinki Declaration and the protocol was approved by the University of Pecs Institutional Ethical Review Board (Number: 8672-PTE 2021). Written informed consent was obtained from all study subjects.

A complete ophthalmic examination was carried out on every study subject including visual acuity, intraocular pressure, slit-lamp examination with dilated fundus examination, and in vivo confocal microscopy (Heidelberg Retina Tomograph II Rostock Cornea Module; Heidelberg Engineering GmbH, Heidelberg, Germany). The IVCM exam utilized topical tetracaine hydrochloride 0.4%, a sterile corneal cap of polymethylmethacrylate (Tomo-Cap–Heidelberg Engineering GmbH) filled with a dense Vidisic gel (Bausch & Lomb, Berlin, Germany). The cap was positioned over the objective lens. A single eye for each participant was randomly selected to undergo statistical analysis.

All study subjects underwent in vivo confocal microscopy of all corneal layers as described previously^6^. Three good quality images of the subbasal nerve plexus were selected in three different areas of the central cornea and they were analyzed with ACCMetrics software V3 (University of Manchester, Manchester, UK)^15–19^. Corneal nerve fiber density (NFD), the number of nerve fibers/mm^2^; nerve branch density (NBD), the number of primary branch points on the main nerve fibers/mm^2^; nerve fiber length (NFL), the total length of nerves mm/mm^2^; nerve fiber total branch density (TBD), the total number of branch points/mm^2^, nerve fiber area (NFA), the total nerve fiber area mm^2^/mm^2^; nerve fiber width (NFW), the average nerve fiber width mm/mm^2^ and fractal dimension (FD) were evaluated.

Dendritic cell (DC) size was measured on IVCM images by using the Threshold Function of ImageJ software (http://imagej.nih.gov/ij/; National Institutes of Health, Bethesda, MD, USA). The size of all DCs in three images for each subject were analyzed. Mature DCs with branches and immature DCs without branches were included in the cell count and cell area measurements.

All IVCM examinations were acquired by two experienced examiners (ZK, KN). The image selection and analysis for the IVCM were carefully reviewed by two independent masked examiners (ZK, ES). Low-quality IVCM images or presence of any motion artifacts were excluded from the analysis.

## Statistical analysis

Data were analyzed using the SPSS Statistics 27.0 (IBM Corp., Armonk, NY) and Prism 9.4.1 for macOS (GraphPad Software, San Diego, CA, USA). For each data set, mean, standard deviation (SD) and 95% confidence interval (95% CI) for the mean were calculated. Among the three study groups, variables were compared using analysis of variance (ANOVA) with post-hoc Tukey test. A P value below 0.05 was considered statistically significant.

## Results

This study comprised 29 eyes of 29 vaccinated patients (10 males and 19 females, mean age: 36.66 ± 12.25 years, range 23–51 years) after COVID-19 infection (VCoV group), 28 eyes of 28 non-vaccinated patients (13 males and 15 females, mean age: 42.14 ± 14.17 years, range 25–65 years) after COVID-19 infection (NVCoV group) and 25 eyes of 25 healthy controls (12 males and 13 females, mean age: 47.52 ± 18.45 years, range 22–67 years). No significant difference was found between the three groups regarding age and sex (P = 0.101).

**Figure 1:**
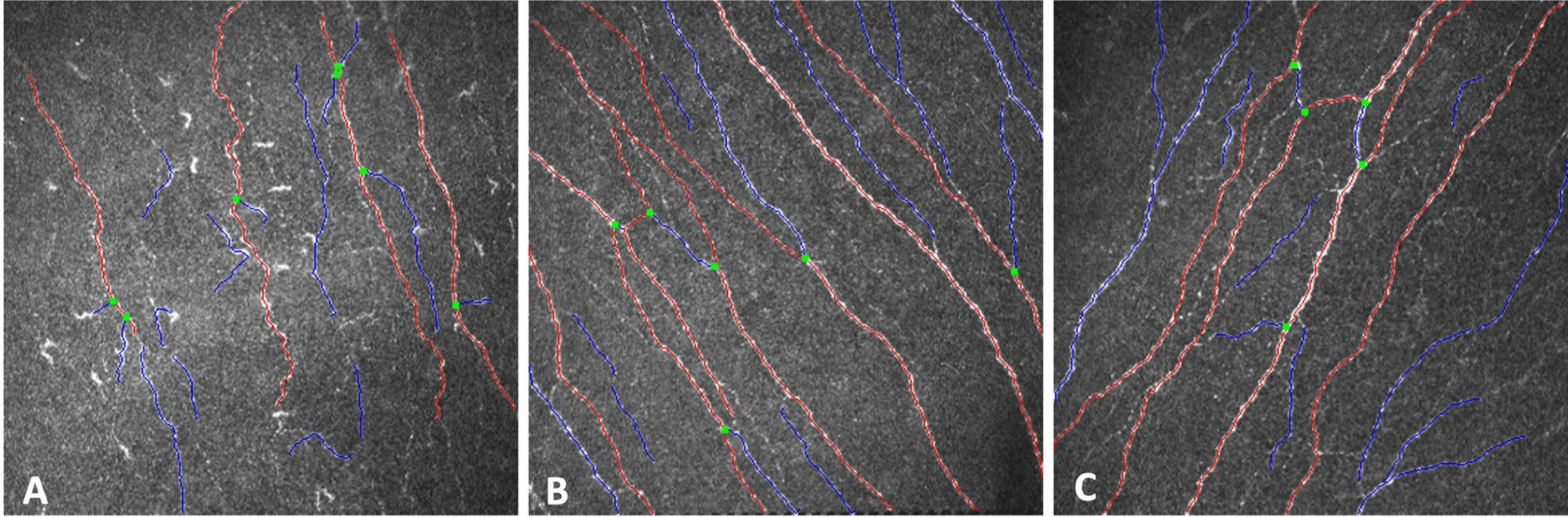
Images of the corneal subbasal nerves analyzed by ACCMetrics software (red: fiber, blue: branch, green: branch point). (**A**) Nerve fiber morphology and scattered dendritic cells of a 49-year-old unvaccinated COVID-19 patient. (**B**) Nerve fiber morphology of a 48-year-old vaccinated COVID-19 patient. (**C**) Nerve fiber morphology of a 50-year-old healthy individual.

Significantly higher corneal nerve fiber density (P < 0.001), nerve branch density (P < 0.001), nerve fiber length (P < 0.001), total branch density (P = 0.007), nerve fiber area (P = 0.001) and fractal dimension (P < 0.001) values were observed in VCoV group compared to NVCoV group (Table 1, Fig. 1). Nerve fiber width was slightly lower in VCoV patients (P = 0.040). There was no statistically significant difference in any nerve fiber morphology parameters between VCoV and healthy individuals (P = 0.435–0.898) (Table 2). Every nerve fiber morphology value was significantly higher in healthy individuals when compared to the NVCoV group except the nerve fiber width (P = 0.378) (Table 3).

**Table 1:** Corneal nerve fiber morphology in vaccinated COVID-19 patients compared to unvaccinated COVID-19 patients.

	Vaccinated COVID-19 patients^§^	Unvaccinated COVID-19 patients^§^	P*
Nerve branch density (No/mm^2^)	23.382 ± 16.331 (17.170–29.594)	8.819 ± 8.595 (5.486–12.151)	< 0.001
Nerve fiber area (mm^2^/mm^2^)	0.006 ± 0.002 (0.005–0.007)	0.004 ± 0.002 (0.003–0.004)	0.001
Nerve fiber density (No/mm^2^)	19.377 ± 8.824 (16.021–22.734)	9.461 ± 5.327 (7.395–11.526)	< 0.001
Nerve fiber length (mm/mm^2^	12.889 ± 4.164 (11.305–14.473)	7.826 ± 2.646 (6.80–8.852)	< 0.001
Nerve fiber width (mm/mm^2^)	0.021 ± 0.002 (0.020–0.022)	0.022 ± 0.002 (0.021–0.023)	0.040
Nerve fiber total branch density (No/mm^2^)	39.204 ± 23.320 (30.333–48.074)	21.12 ± 15.085 (15.271–26.969)	0.007
Fractal dimension	1.470 ± 0.046 (1.453–1.488)	1.369 ± 0.10 (1.331–1.407)	< 0.001
Dendritic cell density (cells/mm^2^)	60.646 ± 58.852 (43.539–77.754)	97.890 ± 95.636 (78.225–117.556)	0.468
Dendritic cell area (µm^2^)	40.266 ± 22.010 (38.640–41.893)	36.288 ± 15.703 (35.155–37.421)	< 0.001

^**§**^Mean ± standard deviation (95% confidence interval).

*Post-hoc Tukey test.

**Table 2:** Corneal nerve fiber morphology in vaccinated COVID-19 patients compared to healthy controls.

	Vaccinated COVID-19 patients^§^	Healthy controls^§^	P*
Nerve branch density (No/mm^2^)	23.382 ± 16.331 (17.170–29.594)	19.895 ± 12.323 (14.808–24.981)	0.725
Nerve fiber area (mm^2^/mm^2^)	0.006 ± 0.002 (0.005–0.007)	0.00524 ± 0.002 (0.004–0.006)	0.671
Nerve fiber density (No/mm^2^)	19.377 ± 8.824 (16.021–22.734)	16.999 ± 7.308 (13.982–20.016)	0.677
Nerve fiber length (mm/mm^2^	12.889 ± 4.164 (11.305–14.473)	11.642 ± 3.528 (10.185–13.098)	0.593
Nerve fiber width (mm/mm^2^)	0.021 ± 0.002 (0.020–0.022)	0.022 ± 0.001 (0.021–0.022)	0.549
Nerve fiber total branch density (No/mm^2^)	39.204 ± 23.320 (30.333–48.074)	35.373 ± 22.035 (26.277–44.468)	0.877
Fractal dimension	1.470 ± 0.056 (1.453–1.488)	1.459 ± 0.044 (1.441–1.477)	0.898
Dendritic cell density (cells/mm^2^)	60.646 ± 58.852 (43.539–77.754)	44.277 ± 60.637 (24.505–63.049)	0.435
Dendritic cell area (µm^2^)	40.266 ± 22.010 (38.640–41.893)	35.463 ± 17.573 (33.228–37.439)	0.810

^**§**^Mean ± standard deviation (95% confidence interval).

*Post-hoc Tukey test.

**Table 3:** Corneal nerve fiber morphology in unvaccinated COVID-19 patients compared to healthy controls.

	Unvaccinated COVID-19 patients^§^	Healthy controls^§^	P*
Nerve branch density (No/mm^2^)	8.819 ± 8.595 (5.486–12.151)	19.895 ± 12.323 (14.808–24.981)	0.008
Nerve fiber area (mm^2^/mm^2^)	0.004 ± 0.002 (0.003–0.004)	0.005 ± 0.002 (0.004–0.006)	0.019
Nerve fiber density (No/mm^2^)	9.461 ± 5.327 (7.395–11.526)	16.999 ± 7.308 (13.982–20.016)	0.001
Nerve fiber length (mm/mm^2^	7.826 ± 2.646 (6.80–8.852)	11.642 ± 3.528 (10.185–13.098)	0.001
Nerve fiber width (mm/mm^2^)	0.022 ± 0.002 (0.021–0.023)	0.022 ± 0.001 (0.021–0.022)	0.378
Nerve fiber total branch density (No/mm^2^)	21.12 ± 15.085 (15.271–26.969)	35.373 ± 22.035 (26.277–44.468)	0.037
Fractal dimension	1.369 ± 0.097 (1.331–1.407)	1.459 ± 0.044 (1.441–1.477)	< 0.001
Dendritic cell density (cells/mm^2^)	97.890 ± 95.636 (78.225–117.556)	44.277 ± 60.637 (24.505–63.049)	0.050
Dendritic cell area (µm^2^)	36.288 ± 15.703 (35.155–37.421)	35.463 ± 17.573 (33.228–37.439)	0.004

^**§**^Mean ± standard deviation (95% confidence interval).

*Post-hoc Tukey test.

**Figure 2:**
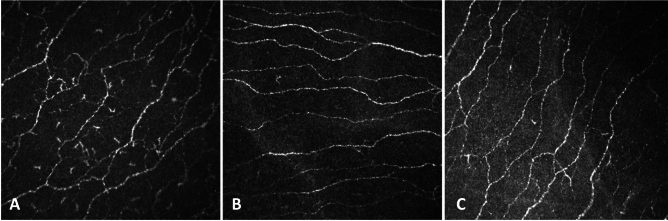
Images of the corneal subbasal nerves and dendritic cells. (**A**) Nerve fiber morphology and scattered dendritic cells of a 29-year-old unvaccinated COVID-19 patient. (**B**) Nerve fiber morphology of a 27-year-old vaccinated COVID-19 patient. (**C**) Nerve fiber morphology of a 27-year-old healthy control.

**Figure 3:**
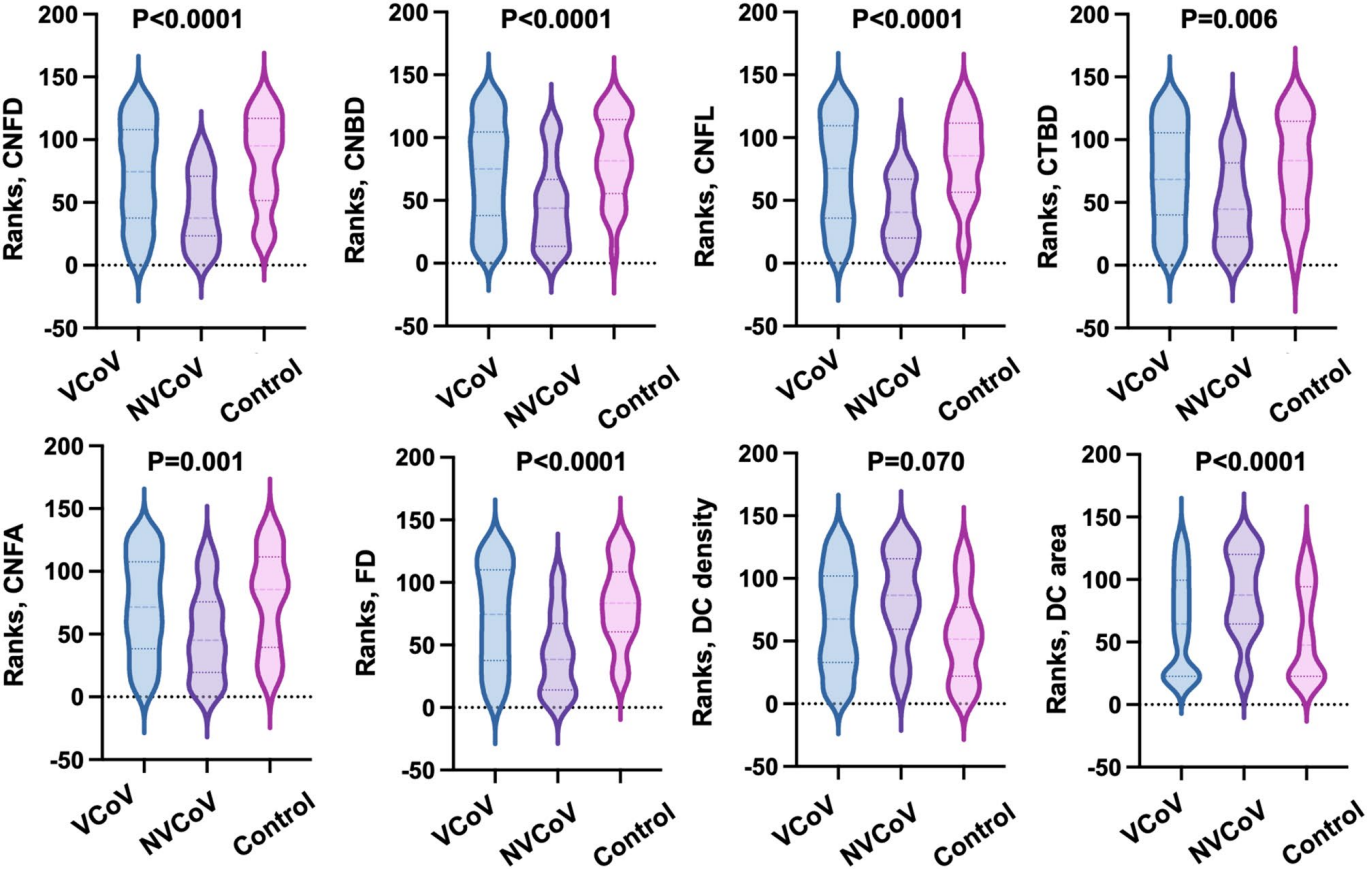
Violin plots of ranks showing the difference among the vaccinated (VCoV), non-vaccinated (NVCoV) COVID-19 groups and control subjects.

Significantly higher density of DC was observed in the NVCoV group compared to the control group (P = 0.05) (Table 1, Fig. 2). There was a statistically significant difference in the size of mature DCs (P < 0.0001) (Fig. 3) but the size of immature DCs did not differ significantly among the 3 groups (P = 0.132). Post-hoc Tukey test revealed significantly higher mature DC area in the NVCoV compared to the VCoV group (P < 0.0001) and in the NVCoV patients compared to the controls (P = 0.004).

## Discussion

SARS-COV-2 virus infection has been described to have significant impact on nearly all organs in the human body, primarily because of the direct influence of the virus and the extensive inflammatory response it triggers^2^. Many ophthalmic complications associated with viral infection have been recorded, including conjunctivitis, keratoconjunctivitis, dry eye disease, episcleritis, acute macular neuroretinopathy, venous and arterial retinal vascular occlusion, optic neuritis, intraretinal hemorrhages, uveitis, and endogenous endophthalmitis^20–23^. Several studies have reported neurological manifestations associated with COVID-19, including effects on the peripheral nervous system. The SARS-CoV-2 virus has been shown to potentially affect nerves and cause various neurological symptoms^24–26^.

Previous authors reported increased corneal DC density and altered nerve fiber morphology in patients with and without long COVID-19. The difference was more pronounced in post-COVID patients with neurological symptoms at 4 weeks^7^. Our previous study demonstrated peripheral small nerve fiber damage in the cornea associated with inflammatory DCs even after mild COVID-19 disease^6^.

To date, there is no direct evidence to support the theory that COVID-19 vaccines have neuroprotective effects. However, the vaccines may indirectly provide some neuroprotective benefits by reducing the risk of COVID-19 infection and its potential neurological complications. While the direct neuroprotective effects of COVID-19 vaccines are not yet fully understood, there is some evidence to suggest that vaccination may be associated with a lower risk of cognitive decline and dementia. A study published in the Journal of Alzheimer's Disease found that individuals who received the influenza vaccine had a lower risk of developing dementia compared to those who did not receive the vaccine. It is important to note that the COVID-19 vaccines are a new development, and long-term studies on their potential neuroprotective effects are still ongoing^27^. However, based on the available evidence, getting vaccinated against COVID-19 is an important step in reducing the risk of infection and potentially reducing the risk of neurological complications.

Besides the benefits of vaccines, several studies reported corneal complications following the administration of the COVID-19 vaccine including corneal graft rejection, herpes zoster ophthalmicus, herpes simplex keratitis, keratolysis and peripheral ulcerative keratitis^28^. Authors explained their findings with the vaccine-induced immunomodulation and that the increased vascular permeability following vaccination impairs the corneal immune privilege^29,30^. Significantly higher DC density of DC was observed in the non-vaccinated group compared to the control group. We observed a statistically significant difference in the size of mature DCs but the size of immature DCs did not differ significantly among the 3 groups. The area of mature DCs was the greatest in the non-vaccinated group followed by the vaccinated COVID-19 group, and the difference between the three groups was statistically significant. A previous case report demonstrated a highly elevated mature DC density during COVID-19 infection (237.5 ± 32.13 cells/mm^2^) and activated DC clumps around corneal infiltrates as a result of an immune-mediated mechanism^31^.

The present investigation has certain limitations that should be taken into consideration when interpreting the outcomes. First, due to the low number of cases, we were unable to examine the differences between the vaccinations, and most of the study participants received not only mRNA-based vaccines but also vector-based and attenuated pathogen-containing vaccines or a combination of them. While a larger study population would always be advantageous, our numbers are similar to previously published cross-sectional comparative investigations^3,5–7^. Similarly powered studies support the relevance of our results. Second, since the SARS-CoV-2 virus changes over time causing various disease types with different severities, the included patients might have been affected by multiple virus variants. It should be emphasized that the vaccine is not neuroprotective in the absence of COVID-19 infection. However, in our study, a reduction in neurodegenerative complications was observed in vaccinated patients compared to unvaccinated patients, possibly due to a milder course of infection. Third, while our study identified statistically significant differences in corneal nerve fiber morphology between vaccinated and non-vaccinated individuals after COVID-19, it is crucial to acknowledge the substantial overlap on the graphs. This overlap suggests that despite the observed differences, the distributions of the nerve plexus parameters in the study groups largely coincide. Consequently, the clinical significance of these findings may be limited, as the overlapping spread indicates that individual variations within each group are considerable.

Our study investigated the role of the vaccine against COVID-19 in terms of the subbasal nerve fibers of the cornea, thus indirectly the peripheral nervous system. Our findings showed that corneal nerve fiber density, nerve branch density, nerve fiber length, nerve fiber total branch density, nerve fiber area and width was increased among the vaccinated subjects compared to the unvaccinated COVID-19 group. Corneal dendritic cell density and area were elevated in the unvaccinated group compared to the vaccinated patients within six months after the infection. In summary, our results suggest that SARS-CoV-2 vaccination may have a protective effect against the complications of COVID-19 disease on the corneal subbasal nerve fibers.

## Data Availability

The datasets used and/or analysed during the current study available from the corresponding author on reasonable request.

## References

[1] Chou, S. H. *et al.* Global Incidence of neurological manifestations among patients hospitalized with COVID-19-A report for the GCS-neuroCOVID consortium and the ENERGY consortium. *JAMA Netw. Open* **4**(5), e2112131. 10.1001/jamanetworkopen.2021.12131 (2021). 33974053 10.1001/jamanetworkopen.2021.12131 PMC8114143

[2] van Eijk, L. E. *et al.* COVID-19: Immunopathology, pathophysiological mechanisms, and treatment options. *J. Pathol.* **254**(4), 307–331. 10.1002/path.5642 (2021). 33586189 10.1002/path.5642 PMC8013908

[3] Mirza, E., Belviranli, S., Gundogan, A. O., Adam, M. & Oltulu, R. Quantitative assessment of the effect of SARS-CoV-2 on the corneal sub-basal nerve plexus of post-COVID-19 patients using in vivo confocal microscopy. *Eye (Lond.)* **37**(4), 660–664. 10.1038/s41433-022-02018-1 (2023). 35322211 10.1038/s41433-022-02018-1 PMC8941366

[4] Midena, E. *et al.* Small fiber peripheral alterations following COVID-19 detected by corneal confocal microscopy. *J. Pers. Med.* **12**(4), 563. 10.3390/jpm12040563 (2022). 35455679 10.3390/jpm12040563 PMC9030195

[5] Barros, A. *et al.* Small fiber neuropathy in the cornea of Covid-19 patients associated with the generation of ocular surface disease. *Ocul. Surf.* **23**, 40–48. 10.1016/j.jtos.2021.10.010 (2022). 34781021 10.1016/j.jtos.2021.10.010 PMC8588585

[6] Kolkedi, Z., Csutak, A. & Szalai, E. Corneal cellular and neuroinflammatory changes after SARS-CoV-2 infection. *Cornea* **41**(7), 879–885. 10.1097/ICO.0000000000003018 (2022). 35349500 10.1097/ICO.0000000000003018

[7] Bitirgen, G. *et al.* Corneal confocal microscopy identifies corneal nerve fiber loss and increased dendritic cells in patients with long COVID. *Br. J. Ophthalmol.* **106**(12), 1635–1641. 10.1136/bjophthalmol-2021-319450 (2022). 34312122 10.1136/bjophthalmol-2021-319450 PMC8359871

[8] Baden, L. R. *et al.* Efficacy and safety of the mRNA-1273 SARS-CoV-2 vaccine. *N. Engl. J. Med.* **384**(5), 403–416. 10.1056/NEJMoa2035389 (2021). 33378609 10.1056/NEJMoa2035389 PMC7787219

[9] Polack, F. P. *et al.* Safety and efficacy of the BNT162b2 mRNA Covid-19 vaccine. *N. Engl. J. Med.* **383**(27), 2603–2615. 10.1056/NEJMoa2034577 (2020). 33301246 10.1056/NEJMoa2034577 PMC7745181

[10] Voysey, M. *et al.* Safety and efficacy of the ChAdOx1 nCoV-19 vaccine (AZD1222) against SARS-CoV-2: An interim analysis of four randomised controlled trials in Brazil, South Africa, and the UK. *Lancet* **397**(10269), 99–111. 10.1016/S0140-6736(20)32661-1 (2021). 33306989 10.1016/S0140-6736(20)32661-1 PMC7723445

[11] Sadoff, J. *et al.* Safety and efficacy of single-dose Ad26.COV2.S vaccine against Covid-19. *N. Engl. J. Med.* **384**(23), 2187–2201. 10.1056/NEJMoa2101544 (2021). 33882225 10.1056/NEJMoa2101544 PMC8220996

[12] Logunov, D. Y. *et al.* Safety and immunogenicity of an rAd26 and rAd5 vector-based heterologous prime-boost COVID-19 vaccine in two formulations: Two open, non-randomised phase 1/2 studies from Russia. *Lancet* **396**(10255), 887–897. 10.1016/S0140-6736(20)31866-3 (2020). 32896291 10.1016/S0140-6736(20)31866-3 PMC7471804

[13] Al Kaabi, N. *et al.* Effect of 2 inactivated SARS-CoV-2 vaccines on symptomatic COVID-19 infection in adults: A randomized clinical trial. *JAMA* **326**(1), 35–45. 10.1001/jama.2021.8565 (2021). 34037666 10.1001/jama.2021.8565 PMC8156175

[14] Villadiego, J. *et al.* Full protection from SARS-CoV-2 brain infection and damage in susceptible transgenic mice conferred by MVA-CoV2-S vaccine candidate. *Nat. Neurosci.* **26**(2), 226–238. 10.1038/s41593-022-01242-y (2023). 36624276 10.1038/s41593-022-01242-y

[15] Dabbah, M. A., Graham, J., Petropoulos, I. N., Tavakoli, M. & Malik, R. A. Automatic analysis of diabetic peripheral neuropathy using multi-scale quantitative morphology of nerve fibers in corneal confocal microscopy imaging. *Med. Image Anal.* **15**(5), 738–747. 10.1016/j.media.2011.05.016 (2011). 21719344 10.1016/j.media.2011.05.016

[16] Dabbah, M. A., Graham, J., Petropoulos, I., Tavakoli, M. & Malik, R. A. Dual-model automatic detection of nerve-fibers in corneal confocal microscopy images. *Med. Image Comput. Comput. Assist. Interv.* **13**(1), 300–307. 10.1007/978-3-642-15705-9_37 (2010). 20879244 10.1007/978-3-642-15705-9_37 PMC3066470

[17] Chen, X. *et al.* An automatic tool for quantification of nerve fibers in corneal confocal microscopy images. *IEEE Trans. Bio Med. Eng.* **64**(4), 786–794. 10.1109/TBME.2016.2573642 (2017). 10.1109/TBME.2016.2573642 PMC5512547 27295646

[18] Petropoulos, I. N. *et al.* Rapid automated diagnosis of diabetic peripheral neuropathy with in vivo corneal confocal microscopy. *Invest. Ophthalmol. Vis. Sci.* **55**(4), 2071–2078. 10.1167/iovs.13-13787 (2014). 24569580 10.1167/iovs.13-13787 PMC3979234

[19] Petropoulos, I. N. *et al.* Repeatability of in vivo corneal confocal microscopy to quantify corneal nerve morphology. *Cornea* **32**(5), e83–e89. 10.1097/ICO.0b013e3182749419 (2013). 23172119 10.1097/ICO.0b013e3182749419

[20] Al-Namaeh, M. Ocular manifestations of COVID-19. *Ther. Adv. Ophthalmol.* **14**, 25158414221083376. 10.1177/25158414221083374 (2022). 35434520 10.1177/25158414221083374 PMC9008819

[21] Bertoli, F. *et al.* Ocular findings in COVID-19 patients: A review of direct manifestations and indirect effects on the eye. *J. Ophthalmol.* **2020**, 4827304. 10.1155/2020/4827304 (2020). 32963819 10.1155/2020/4827304 PMC7491448

[22] Domínguez-Varela, I. A. *et al.* COVID-19 and the eye: A review. *Infect. Dis. (London, England)* **53**(6), 399–403. 10.1080/23744235.2021.1882697 (2021). 10.1080/23744235.2021.1882697 33566704

[23] Dong, J., Chen, R., Zhao, H. & Zhu, Y. COVID-19 and ocular complications: A review of ocular manifestations, diagnostic tools, and prevention strategies. *Advances in Ophthalmol. Pract. Res.* **3**(1), 33–38. 10.1016/j.aopr.2022.11.001 (2023). 36471811 10.1016/j.aopr.2022.11.001 PMC9714126

[24] Harapan, B. N. & Yoo, H. J. Neurological symptoms, manifestations, and complications associated with severe acute respiratory syndrome coronavirus 2 (SARS-CoV-2) and coronavirus disease 19 (COVID-19). *J. Neurol.* **268**(9), 3059–3071. 10.1007/s00415-021-10406-y (2021). 33486564 10.1007/s00415-021-10406-y PMC7826147

[25] Aghagoli, G. *et al.* Neurological Involvement in COVID-19 and potential mechanisms: A review. *Neurocrit. Care* **34**(3), 1062–1071. 10.1007/s12028-020-01049-4 (2021). 32661794 10.1007/s12028-020-01049-4 PMC7358290

[26] Montalvan, V., Lee, J., Bueso, T., De Toledo, J. & Rivas, K. Neurological manifestations of COVID-19 and other coronavirus infections: A systematic review. *Clin. Neurol. Neurosurg.* **194**, 105921. 10.1016/j.clineuro.2020.105921 (2020). 32422545 10.1016/j.clineuro.2020.105921 PMC7227498

[27] Bukhbinder, A. S. *et al.* Risk of Alzheimer’s disease following influenza vaccination: A claims-based cohort study using propensity score matching. *J. Alzheimer’s Dis. JAD* **88**(3), 1061–1074. 10.3233/JAD-220361 (2022). 35723106 10.3233/JAD-220361 PMC9484126

[28] Huang, L. Y. *et al.* Corneal complications after COVID-19 vaccination: A systemic review. *J. Clin. Med.* **11**(22), 6828. 10.3390/jcm11226828 (2022). 36431307 10.3390/jcm11226828 PMC9698276

[29] Rehman, O., Arya, S. K., Jha, U. P., Nayyar, S. & Goel, I. Herpes zoster ophthalmicus after COVID-19 vaccination: Chance occurrence or more?. *Cornea* **41**(2), 254–256. 10.1097/ICO.0000000000002881 (2022). 34690265 10.1097/ICO.0000000000002881

[30] Steinemann, T. L., Koffler, B. H. & Jennings, C. D. Corneal allograft rejection following immunization. *Am. J. Ophthalmol.* **106**(5), 575–578. 10.1016/0002-9394(88)90588-0 (1988). 3056015 10.1016/0002-9394(88)90588-0

[31] Mangan, M. S., Yildiz-Tas, A., Yildiz, M. B., Yildiz, E. & Sahin, A. In vivo confocal microscopy findings after COVID-19 infection. *Ocul. Immunol. Inflamm.* **30**, 1866–1868. 10.1080/09273948.2021.1966051 (2022). 34383621 10.1080/09273948.2021.1966051

